# Contemporary Disengagement From Antiretroviral Therapy in the Western Cape, South Africa: A Cross‐Sectional Study

**DOI:** 10.1002/jia2.70124

**Published:** 2026-05-18

**Authors:** Jonathan Euvrard, Claire Marriott Keene, Erin von der Heyden, Meg Osler, David Pienaar, Hassan Mahomed, Graeme Meintjes, Mary‐Ann Davies, Andrew Boulle

**Affiliations:** ^1^ Centre for Integrated Data and Epidemiological Research School of Public Health University of Cape Town Cape Town South Africa; ^2^ Department of Health and Wellness Provincial Government of the Western Cape Cape Town South Africa; ^3^ NDM Centre for Global Health Research Nuffield Department of Medicine University of Oxford Oxford UK; ^4^ Division of Health Systems and Public Health Department of Global Health Stellenbosch University Stellenbosch South Africa; ^5^ Department of Medicine University of Cape Town Cape Town South Africa; ^6^ Institute of Infectious Disease and Molecular Medicine University of Cape Town Cape Town South Africa; ^7^ Blizard Institute Faculty of Medicine and Dentistry Queen Mary University of London London UK

**Keywords:** antiretroviral therapy (ART), engagement, HIV care continuum, HIV, retention, routine health data

## Abstract

**Introduction:**

South Africa has the largest antiretroviral therapy (ART) programme in the world, with universal access available through the public health system. Yet, gaps in coverage persist. In the Western Cape (WC), an estimated 200,000 people living with HIV are not currently on ART—many of whom are known to the health services. Exploring how people who are not on ART differ from those who are on ART may help guide more effective strategies for re‐engagement and retention in care.

**Methods:**

We conducted a cross‐sectional analysis of routine person‐level data from the WC Provincial Health Data Centre, including adults (≥15 years) known to be living with HIV who accessed public services between October 2022 and September 2024. ART status was inferred from visit and dispensing records. Relative risks (RRs) of current disengagement were estimated using multivariable log‐binomial regression on 25 imputed data sets, adjusting for sex, age, years since diagnosis, diagnosis setting and baseline CD4 count.

**Results:**

Of 494,071 adults included, 131,368 (27%) were currently disengaged from ART. Those at elevated risk included men (aRR 1.20, 95% CI 1.19–1.21), younger people aged 15–24 years (aRR 1.54, 95% CI 1.51–1.57), those with CD4 >500 cells/mm^3^ at diagnosis (aRR 1.26, 95% CI 1.24–1.28) and individuals diagnosed in hospital (aRR 1.41, 95% CI 1.39–1.43) or during pregnancy (aRR 1.20, 95% CI 1.18–1.22). However, the majority of those disengaged were not from these groups, proportionally representing the underlying population living with HIV. Model discrimination was poor (AUC 0.614), indicating that these characteristics do not reliably identify those disengaged.

**Conclusions:**

Most disengaged individuals are from larger, lower‐risk demographic groups and would be missed by interventions targeting higher‐risk demographics. Whole‐population strategies that address common barriers to retention through more inclusive, person‐centred care offer the greatest potential to improve ART coverage.

## 1 Introduction

In 2023, among 7.7 million people living with HIV in South Africa, 5.9 million (77%) were accessing antiretroviral therapy (ART) [1]. In the Western Cape (WC), ART coverage among all those living with HIV increased from 64% in 2020 to over 67% in 2024 [2]. Since the introduction of universal test‐and‐treat guidelines in 2016, all people living with HIV have been eligible for lifelong ART, which can lead to a healthy, near‐normal life expectancy and prevent HIV transmission [3, 4]. ART is widely available and free at public healthcare clinics in South Africa [5].

To maximize the individual and public health benefits of ART, further efforts are needed to reach the estimated 200,000 people living with HIV in the WC who are not currently on ART [6]. In 2025, the South African Department of Health launched the *Close The Gap* campaign with the aim to initiate and reinitiate an additional 1.1 million people on ART in South Africa [7].

Disengagement from care is often highlighted as a key barrier to ART coverage [8, 9], and recent evidence suggests that most people living with HIV who are not on ART are known to the health system [10]. A recent WC study using routine health data found that at least 70% of those estimated as not currently receiving ART had prior contact with health services, suggesting that interventions may benefit from prioritizing re‐engagement of this group rather than only focusing on those who have not had contact with the health services [6].

This study examines how health system factors and patient characteristics evident in routine health data are associated with current disengagement from ART, providing novel insights from a large population cohort that may inform the targeting of interventions aimed at improving retention, promoting re‐engagement and thereby increasing ART coverage.

## 2 Methods

### 2.1 Study Population and Data Sources

For this study, we conducted a cross‐sectional analysis of routine person‐level data collected by public health services. We included adults 15 years and older known to be living with HIV who accessed public healthcare services in WC in the 2 years prior to database closure on 30 September 2024. We excluded people with missing demographics.

The WC Provincial Health Data Centre (PHDC) has been described in detail elsewhere [11, 12, 13]. Briefly, a unique patient identifier is leveraged to harmonize linked patient‐level electronic data from all public health information systems in WC, including laboratory, pharmacy and clinical systems and electronic disease registers. All public healthcare facilities in the province, including over 300 clinics and hospitals, contributed to this linked data set.

This study was approved by the WC Department of Health and Wellness (WC_202107_031) and the University of Cape Town Human Research Ethics Committees (HREC 379/2021). The requirement for individual informed consent was waived as this was a secondary analysis of de‐identified routine data.

### 2.2 Key Variables

We included in this analysis the following key variables as they were readily identifiable in the routine data. *HIV diagnosis date* in the PHDC was digitally recorded using International Statistical Classification of Diseases and Related Health Problems (ICD) codes or an electronic disease register, or inferred from other digitized evidence such as laboratory tests or ART dispensing. *Diagnosis setting* was assigned hierarchically: hospital diagnoses first, followed by those diagnosed through the vertical transmission prevention (VTP) programme—defined as diagnosis during a known pregnancy—then diagnoses at the time of active tuberculosis (TB). Remaining diagnoses were classified under general Primary Healthcare (PHC) services. *Diagnosis CD4* was the first result from a sample taken between 12 months before and 2 weeks after the first evidence of diagnosis, categorized as 0–200, 201–350, 351–500 and >500 cells/mm^3^. *Years since diagnosis* was the time from diagnosis to database closure. *ART initiation date* was recorded in an electronic register or inferred from ART dispensing. *ART status* was assigned based on the last visit date plus days of ART dispensed; individuals were classified as on ART if that date, plus 90 days, was on or after database closure (30 September 2024) or as currently disengaged if not (i.e. 90 days with no ART in hand).

### 2.3 Statistical Analysis

We compared people who were currently disengaged to those who were on ART. Within each group, we described sex, age at diagnosis, diagnosis CD4, diagnosis setting, years since diagnosis and age at database closure (Table 1). We reported medians and interquartile ranges (IQRs) for continuous variables by ART status.

**TABLE 1:** Characteristics of adults living with HIV in the Western Cape by current antiretroviral therapy status.

	On ART		Disengaged (not on ART)		Total	
**Sex**						
Female	250,634	69%	86,806	66%	337,440	68%
Male	112,069	31%	44,562	34%	156,631	32%
**Age at diagnosis**						
*Median, IQR*	*32*	*26*−*38*	*30*	*24*−*37*	*31*	*25*−*38*
<15 years	7742	2%	3210	2%	10,952	2%
15−24 years	69,873	19%	34,833	27%	104,706	21%
25−34 years	155,277	43%	54,105	41%	209,382	42%
35−44 years	89,942	25%	25,958	20%	115,900	23%
45−54 years	31,507	9%	9522	7%	41,029	8%
≥ 55 years	8362	2%	3740	3%	12,102	2%
**Diagnosis CD4**						
*Median, IQR*	*292*	*177−430*	*318*	*202−484*	*298*	*183−445*
0−200 cells/mm^3^	75,061	21%	21,829	17%	96,890	20%
201−350 cells/mm^3^	91,215	25%	29,323	22%	120,538	24%
351−500 cells/mm^3^	43,607	12%	16,894	13%	60,501	12%
500+ cells/mm^3^	46,020	13%	20,656	16%	66,676	13%
Missing	106,800	29%	42,666	32%	149,466	30%
**Diagnosis setting**						
Hospital	27,891	8%	14,549	11%	42,440	9%
PHC VTP	32,278	9%	14,671	11%	46,949	10%
PHC TB	42,747	12%	14,741	11%	57,488	12%
PHC other	259,787	72%	87,407	67%	347,194	70%
**Years since diagnosis**						
*Median, IQR*	*9*	*5*−*13*	*7*	*3*−*11*	*8*	*4*−*12*
<5 years	94,570	26%	47,123	36%	141,693	29%
5−10 years	118,316	33%	45,578	35%	163,894	33%
>10 years	149,817	41%	38,667	29%	188,484	38%
**Age at database closure**						
*Median, IQR*	*41*	*34*−*48*	*37*	*31*−*45*	*40*	*33*−*47*
15−24 years	18,739	5%	10,127	8%	28,866	6%
25−34 years	79,701	22%	42,016	32%	121,717	25%
35−44 years	132,648	37%	45,736	35%	178,384	36%
45−54 years	92,060	25%	22,289	17%	114,349	23%
≥ 55 years	39,555	11%	11,200	9%	50,755	10%
**Total**	**362,703**		**131,368**		**494,071**	

*Note*: Columns represent absolute numbers and percentages within the group, Totals are in bold, italics for medians and interquartile ranges (IQR).

We calculated the proportion of people with a missing diagnosis CD4 and examined the associations between missingness and covariates (Table S1). Using those covariates and the outcome, we used ordered logistic regression to impute 25 data sets with complete baseline CD4 data. A log binomial regression model with robust standard errors was used to estimate crude and adjusted risk ratios (RRs) between sex, diagnosis CD4 category, diagnosis setting, years since diagnosis and age category at database closure on the relative risk of current disengagement. Estimates and confidence intervals (CIs) were pooled using Rubin's rules for imputed data analyses. We assessed the model's discriminative ability using the area under the receiver operating characteristic curve (AUC), calculated separately within each imputed data set and then pooled. Data analysis was conducted using STATA 14 (STATA Corporation).

## 3 Results

### 3.1 Patient Characteristics

We enumerated person‐level data for 494,892 adults 15 years and older, known to be living with HIV, who last interacted with public healthcare services in WC between 1 October 2022 and 30 September 2024. We excluded 821 people with missing demographics (sex unavailable), leaving 494,071 people included in the analysis (Figure S1).

Our study included 337,440 (68%) women and 156,631 (32%) men (Table 1). Participants had a median age at database closure of 40 years (IQR 33–47). Most people (70%) were diagnosed in general PHC services, with 9% diagnosed in hospital, 10% through the VTP programme and 12% through the TB programme. Almost a third (29%) were diagnosed within the past 5 years, another third (33%) between 5 and 10 years ago, and the rest (38%) more than 10 years ago. CD4 counts at diagnosis were present for 70% of people, among whom the median CD4 count was 298 cells/mm^3^ (IQR 183–445).

### 3.2 Disengaged

**FIGURE 1:**
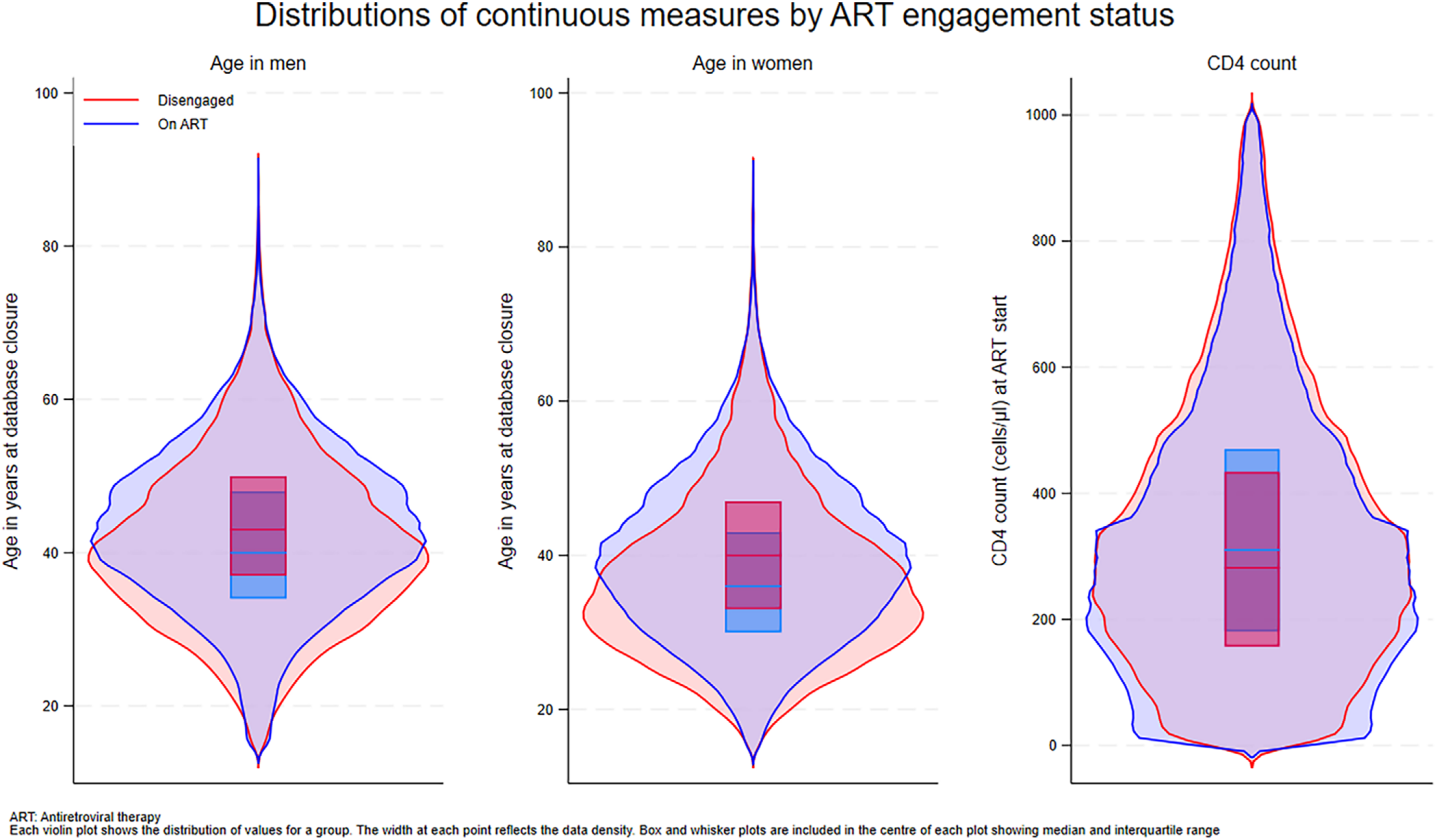
Violin plots comparing the distribution of continuous variables among those currently disengaged (not on ART) to those on ART.

Of 494,071 adults included in the analysis, 131,368 (27%) were currently disengaged (Table 1). Women represented two‐thirds (66%) of those disengaged and slightly more than two‐thirds (69%) of those on ART. Median age at database closure was slightly lower among those disengaged at 37 years (IQR 31–45) compared to those on ART at 41 years (IQR 34–48) (Figure 1). Women aged 25–54 years constituted 72,802 (55%) of 131,368 adults not on ART (Table 2).

**TABLE 2:** Characteristics of adults living with HIV in the Western Cape by current antiretroviral therapy status, shown by combined sex‐age categories.

	On ART		Disengaged (not on ART)		Total	
**Sex**						
Female	250,634	69%	86,806	66%	337,440	68%
**Age at database closure**						
*15−24 years*	14,363	4%	7868	6%	22,231	4%
*25−34 years*	63,289	17%	31,847	24%	95,136	19%
*35−44 years*	92,311	25%	28,875	22%	121,186	25%
*45−54 years*	56,970	16%	12,080	9%	69,050	14%
*≥ 55 years*	23,701	7%	6136	5%	29,837	6%
Male	112,069	31%	44,562	34%	156,631	32%
**Age at database closure**						
*15−24 years*	4376	1%	2259	2%	6635	1%
*25−34 years*	16,412	5%	10,169	8%	26,581	5%
*35−44 years*	40,337	11%	16,861	13%	57,198	12%
*45−54 years*	35,090	10%	10,209	8%	45,299	9%
*≥ 55 years*	15,854	4%	5064	4%	20,918	4%
**Total**	**362,703**		**131,368**		**494,071**	

*Note*: Columns represent absolute numbers and percentages of the total.

Adults currently disengaged had higher CD4 counts at diagnosis with a median of 318 cells/mm^3^ (IQR 202–484) compared to those on ART with a median of 292 cells/mm^3^ (IQR 177–430). It was noted that, among both groups, a large proportion of people had CD4 counts below 200 cells/mm^3^ at diagnosis: 17% and 21% among those currently disengaged and on ART, respectively.

Compared to those on ART, disengaged individuals were less likely to have been diagnosed in PHC clinics (67% vs. 72%) and more likely through the VTP programme (11% vs. 9%), while proportions were similar for hospital (11%) and TB programme diagnoses (11% vs. 12%). Time since diagnosis also differed: among those disengaged, 36% were diagnosed in the past 5 years, 35% 5−10 years ago and 29% more than 10 years ago, compared to 26%, 33% and 41%, respectively, for those on ART.

### 3.3 Associations With Current Disengagement

#### 3.3.1 Univariable Analysis

We estimated the crude relative risk of current disengagement (Table 3). Men were at higher risk of current disengagement than women, relative risk (RR) 1.11 (95% CI 1.10–1.12). Those in higher CD4 categories at diagnosis were at higher risk of current disengagement compared to those with a diagnosis CD4 of less than 200 cells/mm^3^, with RRs between 1.10 for those with CD4 201–350 and 1.37 for those with CD4>500 cells/mm^3^.

**TABLE 3:** Associations with current disengagement with 95% confidence intervals (95% CI).

	Total	Disengaged (not on ART)	RR (95% CI)	aRR (95% CI)
**Sex**				
Women	337,440	86,806 (26%)		
Men	156,631	44,562 (28%)	1.11 (1.10–1.12)	1.20 (1.19–1.21)
**Diagnosis CD4**				
0−200 cells/mm^3^	96,890	21,829 (23%)		
201−350 cells/mm^3^	120,538	29,323 (24%)	1.10 (1.08–1.11)	1.11 (1.10–1.13)
351−500 cells/mm^3^	60,501	16,894 (28%)	1.25 (1.23–1.27)	1.16 (1.14–1.18)
500+ cells/mm^3^	66,676	20,656 (31%)	1.37 (1.35–1.39)	1.26 (1.24–1.28)
**Diagnosis setting**				
Hospital	42,440	14,549 (34%)	1.36 (1.34–1.38)	1.41 (1.39–1.43)
PHC VTP	46,949	14,671 (31%)	1.24 (1.22–1.26)	1.20 (1.18–1.22)
PHC TB	57,488	14,741 (26%)	1.02 (1.00–1.03)	1.10 (1.09–1.12)
PHC other	347,194	87,407 (25%)		
**Years since diagnosis**				
<5 years	141,693	47,123 (33%)	1.62 (1.60–1.64)	1.36 (1.34–1.38)
5−10 years	163,894	45,578 (28%)	1.36 (1.34–1.37)	1.22 (1.21–1.24)
>10 years	188,484	38,667 (21%)		
**Age at analysis date**				
15−24 years	28,866	10,127 (35%)	1.80 (1.76–1.84)	1.54 (1.51–1.57)
25−34 years	121,717	42,016 (35%)	1.77 (1.75–1.80)	1.56 (1.54–1.58)
35−44 years	178,384	45,736 (26%)	1.32 (1.30–1.33)	1.25 (1.23–1.27)
45−54 years	114,349	22,289 (19%)		
≥ 55 years	50,755	11,200 (22%)	1.13 (1.11–1.16)	1.13 (1.10–1.15)
**Total**	**494,071**			

Abbreviations: aRR, adjusted risk ratio; PHC, primary healthcare; RR, risk ratio; TB, tuberculosis; VTP, vertical transmission prevention.

We estimated the relative risk of current disengagement by diagnosis setting compared to the most common setting, the PHC clinic. Diagnosis in hospital and during pregnancy showed an elevated risk of RR 1.36 (95% CI 1.34–1.38) and 1.24 (95% CI 1.22–1.26), respectively. Those diagnosed more recently showed RR 1.62 (95% CI 1.60–1.64) in the most recent period compared to those diagnosed more than 10 years ago.

#### 3.3.2 Multivariable Analysis

**FIGURE 2:**
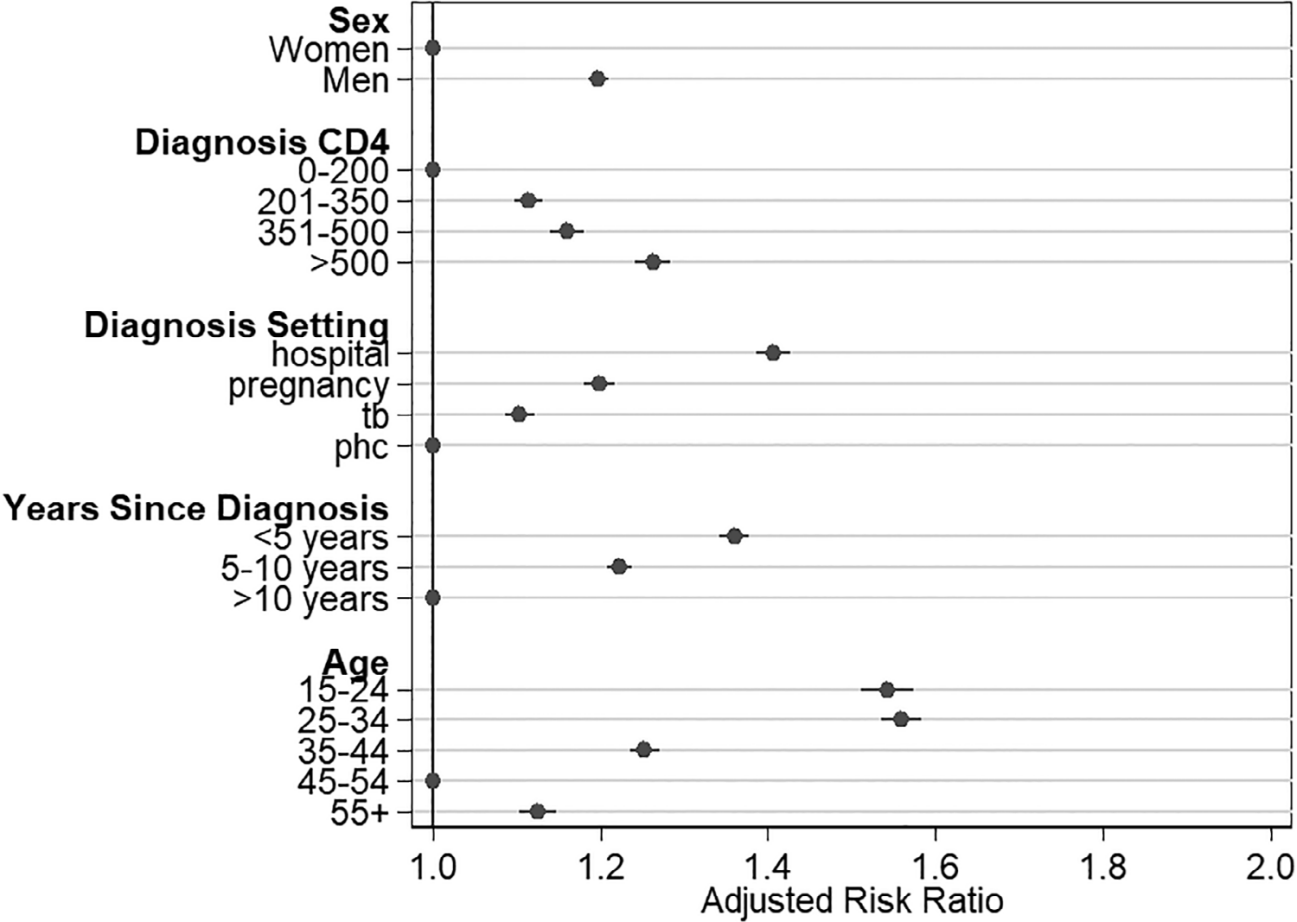
Adjusted associations with current disengagement, with 95% confidence intervals. Abbreviations: PHC, primary healthcare; TB, tuberculosis.

In the adjusted model, men remained at higher risk of current disengagement compared to women, aRR 1.20 (95% CI 1.19–1.21) (Table 3 and Figure 2). Those with higher CD4 counts at HIV diagnosis remained at higher risk of current disengagement compared to those with CD4 counts <200 cells/mm^3^, aRR 1.11–1.26. Diagnosis in hospital and through the VTP programme presented a higher risk than diagnosis in PHC clinics. A recent HIV diagnosis also presented a higher risk than a diagnosis more than 10 years ago, aRR 1.36 (95% CI 1.34–1.38). Current age categories below and above the 45‐ to 54‐year‐old group experienced a higher risk of current disengagement, highest among those 15–24 years old, aRR 1.54 (95% CI 1.51–1.57).

#### 3.3.3 Sensitivity Analysis

The multivariable analysis was reproduced following a complete case analysis approach restricted to 344,605 individuals with a baseline CD4 present, of whom 88,702 (26%) were currently disengaged (Table S2). The baseline characteristics of people included in the complete case analysis and the estimates of adjusted relative risk produced by the complete case model were comparable (Table S3).

#### 3.3.4 Assessing the Model

We did not pursue alternative model specifications or more complex approaches, as the goal was not to optimize prediction accuracy but to assess whether readily available demographic and clinical characteristics provide sufficient discrimination to support targeted re‐engagement strategies.

The pooled AUC of 0.614 indicates poor discrimination [14]. To better understand the practical implications of the model's limited discriminative ability, we estimated the proportion of the population that would need to be targeted (classified as high risk) to identify different proportions of individuals who were disengaged. On average across imputations, identifying half of those disengaged would require classifying 62% of the population as high risk (Table S4). This illustrates that the model does not effectively concentrate risk within a small subgroup, limiting its potential utility for targeting interventions.

## 4 Discussion

This cross‐sectional analysis leveraged a large, population‐wide routine data set to ascertain the treatment status of all adults known to be living with HIV in WC. The adjusted regression model identified a set of modest associations with current disengagement that are consistent with previous studies in South Africa, including higher risk among men, people diagnosed with higher CD4 counts, those diagnosed outside of general primary healthcare services, those more recently diagnosed, and those at both younger and older ages [15, 16]. What this study adds is contemporary, highly precise estimates generated at a population scale, demonstrating that while these associations are statistically robust, their effect sizes are small. This implies that, although identifiable subgroups remain at somewhat higher individual risk of disengagement, their predictive value for distinguishing who is disengaged is limited.

While most studies have shown an association between sex and the risk of current disengagement, the direction of association is not consistent, even across sub‐Saharan Africa. A large systematic review found that, of 28 sub‐Saharan studies included, six studies found that women were at higher risk of disengagement than men [17]. This suggests that sex is more likely a signal for barriers to care that affect men and women in different ways in different contexts, rather than a risk factor in itself.

For each of the described associations in this study, the magnitude of risk was small, and the groups at lower risk represented the largest proportion of people. For example, while men were at slightly higher risk of current disengagement than women, there were almost twice as many women than men currently disengaged, similar to the proportion of women in the population currently on ART. Most of the adults who were identified as disengaged in this population were women aged 25–54 years (55%), because most people living with HIV in this population were women aged 25–54 years (58%).

In total, over a quarter (27%) of the cohort was currently disengaged from care. This included a substantial proportion of individuals in the lower‐risk groups: 26% of women, 23% of those with a CD4 count <200 cells/mm^3^ at diagnosis, 25% of those diagnosed in PHC services, 21% of those diagnosed more than 10 years ago and 19% of those aged 45–54 years. Targeting interventions away from these low‐risk groups is, therefore, unlikely to be effective at improving overall ART coverage, as confirmed by the modest AUC estimate for discriminating between those in and out of care.

This reflects a paradox first described by Geoffrey Rose: although individuals at higher risk have a greater individual likelihood of being disengaged, the majority of those disengaged come from the larger population of those at relatively lower risk [18]. Focusing on high‐risk groups—such as men, young people or those diagnosed outside of general services—is appropriate and efficient at the individual level. But when these efforts come at the expense of addressing the broader population, they risk missing the bulk of people who could benefit from interventions to re‐engage and remain in care [19]. This limits the potential of highly targeted approaches to improve ART coverage at a population level, to improve population health and to decrease HIV transmission, which is particularly important in contexts like South Africa, which are experiencing a mature, generalized HIV epidemic. These findings align with evidence from a systematic review of re‑engagement interventions, which shows that disengaged individuals span all demographic groups and that narrowly targeted strategies have produced mixed effects on re‑engagement and viral suppression [20].

Consistent with this, recent WHO guidance recommends person‑centred, system‑level approaches that reduce barriers for all clients, alongside tracing and differentiated support for those who disengage [21]. A whole‐population approach, one that reduces common barriers to care for everyone—through more person‐centred service delivery and by addressing shared social determinants of health system engagement—can have greater reach. It supports those at highest risk while also benefiting the majority who sit at lower individual risk but still experience obstacles to sustained engagement. In mature, generalized HIV epidemics, this inclusive strategy has the greatest potential to improve population health and reduce transmission overall.

Several limitations may have influenced these findings. This study used routine health data collected for care and service delivery rather than research. While a strength is the use of harmonized patient‐level data across provincial facilities, data missing not at random may have biased estimates of association with current disengagement.

Death was likely under‐ascertained, with unknown variation across subgroups. As individuals known to have died were excluded, unascertained deaths would likely be misclassified as disengaged rather than on ART, similarly with outmigration from the province. If such misclassification disproportionately affected specific groups, it could have inflated their apparent risk of current disengagement.

Despite this, the PHDC's capacity to link data across all public health facilities offers a distinct advantage over analyses limited to single‐site records [22, 23]. Lastly, the diagnosis setting was assigned hierarchically, so missing data defaulted to the general PHC category, used as the reference group, possibly underestimating the impact of other diagnosis settings on current disengagement.

Longitudinal variables were not included in this cross‐sectional analysis. While a history of disengagement has been shown to predict future disengagement, this is a signal for persistent or recurrent barriers to engagement that provides no information on the current barriers themselves [8]. As engagement histories grow longer and more complex, longitudinal studies have used increasingly sophisticated methods such as machine learning and latent class analysis to examine patterns over time [24, 25]. Such approaches were beyond the scope of this study, which sought to review the utility of readily identifiable patient characteristics for improving efficiency of re‐engagement or retention activities.

Future research should focus on identifying and addressing the shared social and structural determinants that influence engagement with HIV services across demographic groups. Evidence from differentiated service delivery, streamlined medication pickup, reduced visit frequency and person‑centred clinic redesign demonstrates that system‑level interventions can improve retention and viral suppression at scale, without reliance on narrowly targeted risk profiling. In high‑volume public sector settings such as the WC, interventions that lower common barriers, such as inflexible service hours, long waiting times, stigma and fragmented care, are more feasible to implement at scale and are likely to have greater population‑level impact than approaches that depend on precise individual risk prediction.

## 5 Conclusions

The WC is home to a large, diverse population of people living with HIV who need lifelong ART. Among those known to the health services, over a quarter were currently disengaged from care. Men, those with higher diagnosis CD4 counts, in younger and older age groups, and diagnosed during pregnancy and in hospital, were at greater relative risk of current disengagement, but the majority of those currently disengaged were outside of these higher risk groups.

These results provide weak signals of underlying barriers to care that disproportionately affect people with identified demographic characteristics. Interventions targeting these groups would miss most people currently disengaged, and risk misinterpreting signals of underlying barriers to care as barriers themselves. Future research and interventions should prioritize the identification and mitigation of barriers to care for the benefit of all.

## Author Contributions

Conceptualization and methodology: JE, CMK, EvdH and AB. Analysis, integration and interpretation: JE, CMK, EvdH, MO, DP, HM, GM, M‐AD and AB. Writing – original draft: JE. Writing – review, editing and approval: JE, CMK, EvdH, MO, DP, HM, GM, M‐AD and AB. All authors have read and approved the final manuscript.

## Funding

GM was supported by the Wellcome Trust (214321/Z/18/Z and 203135/Z/16/Z). For the purpose of open access, the authors have applied a CC BY public copyright licence to any Author Accepted Manuscript version arising from this submission.

AB was supported by the US National Institutes of Health (R01HD080465, U01AI069911), the Bill and Melinda Gates Foundation (1164272, 1191327, INV‐004657, INV‐017293), the Wellcome Trust (203135/Z/16/Z) and the United States Agency for International Development (72067418CA00023).

## Conflicts of Interest

The authors declare no conflicts of interest.

## Supporting information




**Supporting Table S1**: Associations with missing CD4 with 95% confidence intervals (95% CI). Abbreviations: aRR, adjusted risk ratio; PHC, primary healthcare; RR, risk ratio; TB, tuberculosis; VTP, vertical transmission prevention.

**Supporting Table S2**: Characteristics of adults living with HIV in the Western Cape by current antiretroviral therapy (ART) status, restricted to those with CD4 data available (complete case analysis). *Note*: Columns represent absolute numbers and percentages within the group, unless otherwise indicated as median and IQR.

**Supporting Table S3**: Associations with current disengagement with 95% confidence intervals (95% CI), restricted to those with CD4 data available (complete case analysis). Abbreviations: aRR, adjusted risk ratio; PHC, primary healthcare; RR, risk ratio; TB, tuberculosis; VTP, vertical transmission prevention.

**Supporting Table S4**: Estimated proportion of the entire study population (*N* = 494,071) who would need to be targeted (classified as high risk) to identify a target proportion of those disengaged (not on ART), using the full model with multiple imputation for missing CD4 categories.

**Supporting Figure S1**: Enumerated individuals known to the health services to be living with HIV in the Western Cape included in the analysis, excluding those without data on sex at birth.

## Data Availability

The data are not publicly available due to privacy and ethical restrictions. Requests to access data should be directed to the Western Cape Department of Health & Wellness.
