# Supplementary material for: Contemporary Disengagement From Antiretroviral Therapy in the Western Cape, South Africa: A Cross‐Sectional Study
Source: J Int AIDS Soc. 2026 May 18;29(5):e70124. doi: 10.1002/jia2.70124 (PMC13181324; doi:10.1002/jia2.70124)
Supplement: Supplementary file 1 — Supporting Table S1: Associations with missing CD4 with 95% confidence intervals (95% CI). Abbreviations: aRR, adjusted risk ratio; PHC, primary healthcare; RR, risk ratio; TB, tuberculosis; VTP, vertical transmission prevention. [file JIA2-29-e70124-s002.docx]

|  | **Total** | **Missing CD4** | **RR** (95%CI) | **aRR** (95%CI) |
| --- | --- | --- | --- | --- |
| **Sex** |  |  |  |  |
| Women | 337 440 | 104 481 (31%) |  |  |
| Men | 156 631 | 44 985 (29%) | 0,93 (0,92–0,94) | 0,93 (0,92–0,93) |
| **Diagnosis setting** |  |  |  |  |
| Hospital | 42 440 | 17 277 (41%) | 1,25 (1,23–1,26) | 1,20 (1,19–1,22) |
| PHC VTP | 46 949 | 7 861 (17%) | 0,51 (0,50–0,52) | 0,51 (0,50–0,52) |
| PHC TB | 57 488 | 11 187 (19%) | 0,60 (0,59–0,61) | 0,60 (0,59–0,61) |
| PHC other | 347 194 | 113 141 (33%) |  |  |
| **Years since diagnosis** |  |  |  |  |
| <5 years | 141 693 | 38 995 (28%) | 0,77 (0,76–0,77) | 0,74 (0,73–0,75) |
| 5 to 10 years | 163 894 | 42 814 (26%) | 0,73 (0,72–0,74) | 0,72 (0,72–0,73) |
| >10 years | 188 484 | 67 657 (36%) |  |  |
| **Age at analysis date** |  |  |  |  |
| 15-24 years | 10 952 | 6 013 (55%) | 1,13 (1,11–1,15) | 1,21 (1,19–1,23) |
| 25-34 years | 104 706 | 33 412 (32%) | 0,93 (0,92–0,94) | 1,07 (1,06–1,09) |
| 35-44 years | 209 382 | 61 598 (30%) | 0,85 (0,84–0,86) | 0,90 (0,89–0,91) |
| 45-54 years | 114 900 | 33 076 (29%) |  |  |
| ≥ 55 years | 41 029 | 11 219 (27%) | 1,09 (1,07–1,11) | 1,07 (1,06–1,09) |
| **Total** | **494 071** | **149 466 (30%)** |  |  |
