# Supplementary material for: Contemporary Disengagement From Antiretroviral Therapy in the Western Cape, South Africa: A Cross‐Sectional Study
Source: J Int AIDS Soc. 2026 May 18;29(5):e70124. doi: 10.1002/jia2.70124 (PMC13181324; doi:10.1002/jia2.70124)
Supplement: Supplementary file 2 — Supporting Table S2: Characteristics of adults living with HIV in the Western Cape by current antiretroviral therapy (ART) status, restricted to those with CD4 data available (complete case analysis). Note: Columns represent absolute numbers and percentages within the group, unless otherwise indicated as median and IQR. [file JIA2-29-e70124-s003.docx]

|  | **On ART** | | **Disengaged** (not On ART) | | **Total** | |
| --- | --- | --- | --- | --- | --- | --- |
| **Sex** |  |  |  |  |  |  |
| Female | 175 096 | 68% | 57 863 | 65% | 232 959 | 68% |
| Male | 80 807 | 32% | 30 839 | 35% | 111 646 | 32% |
| **Age at diagnosis** |  |  |  |  |  |  |
| *Median, IQR* | *32* | *26-39* | *30* | *24-36* | *31* | *26-38* |
| <15 years | 3 559 | 1% | 1 380 | 2% | 4 939 | 1% |
| 15-24 years | 47 533 | 19% | 23 761 | 27% | 71 294 | 21% |
| 25-34 years | 109 690 | 43% | 37 724 | 43% | 147 414 | 43% |
| 35-44 years | 65 280 | 26% | 17 544 | 20% | 82 824 | 24% |
| 45-54 years | 23 632 | 9% | 6 178 | 7% | 29 810 | 9% |
| ≥ 55 years | 6 209 | 2% | 2 115 | 2% | 8 324 | 2% |
| **Diagnosis CD4** |  |  |  |  |  |  |
| *Median, IQR* | *292* | *177-430* | *318* | *202-484* | *298* | *183-445* |
| 0-200 cells/mm^3^ | 75 061 | 29% | 21 829 | 25% | 96 890 | 28% |
| 201-350 cells/mm^3^ | 91 215 | 36% | 29 323 | 33% | 120 538 | 35% |
| 351-500 cells/mm^3^ | 43 607 | 17% | 16 894 | 19% | 60 501 | 18% |
| 500+ cells/mm^3^ | 46 020 | 18% | 20 656 | 23% | 66 676 | 19% |
| Missing | 66 676 | 26% | 42 666 | 48% | 109 342 | 32% |
| **Diagnosis setting** |  |  |  |  |  |  |
| Hospital | 17 026 | 7% | 8 137 | 9% | 25 163 | 7% |
| PHC VTP | 27 081 | 11% | 12 007 | 14% | 39 088 | 11% |
| PHC TB | 42 747 | 17% | 14 741 | 17% | 57 488 | 17% |
| PHC other | 177 319 | 69% | 56 734 | 64% | 234 053 | 68% |
| **Years since diagnosis** |  |  |  |  |  |  |
| *Median, IQR* | *8* | *5-12* | *7* | *3-10* | *8* | *4-12* |
| <5 years | 70 409 | 28% | 32 289 | 36% | 102 698 | 30% |
| 5 to 10 years | 89 128 | 35% | 31 952 | 36% | 121 080 | 35% |
| >10 years | 96 366 | 38% | 24 461 | 28% | 120 827 | 35% |
| **Age at database closure** |  |  |  |  |  |  |
| *Median, IQR* | *41* | *34-48* | *37* | *31-44* | *40* | *33-47* |
| 15-24 years | 11 978 | 5% | 6 423 | 7% | 18 401 | 5% |
| 25-34 years | 56 558 | 22% | 29 031 | 33% | 85 589 | 25% |
| 35-44 years | 97 585 | 38% | 32 157 | 36% | 129 742 | 38% |
| 45-54 years | 63 338 | 25% | 14 460 | 16% | 77 798 | 23% |
| ≥ 55 years | 26 444 | 10% | 6 631 | 7% | 33 075 | 10% |
| **Total** | **255 903** |  | **88 702** |  | **344 605** |  |
