# Supplementary material for: Contemporary Disengagement From Antiretroviral Therapy in the Western Cape, South Africa: A Cross‐Sectional Study
Source: J Int AIDS Soc. 2026 May 18;29(5):e70124. doi: 10.1002/jia2.70124 (PMC13181324; doi:10.1002/jia2.70124)
Supplement: Supplementary file 3 — Supporting Table S3: Associations with current disengagement with 95% confidence intervals (95% CI), restricted to those with CD4 data available (complete case analysis). Abbreviations: aRR, adjusted risk ratio; PHC, primary healthcare; RR, risk ratio; TB, tuberculosis; VTP, vertical transmission prevention. [file JIA2-29-e70124-s005.docx]

|  | **Total** | **Disengaged** (not On ART) | **RR** (95%CI) | **aRR** (95%CI) |
| --- | --- | --- | --- | --- |
| **Sex** |  |  |  |  |
| Women | 232 959 | 57 863 (25%) |  |  |
| Men | 111 646 | 30 839 (28%) | 1.11 (1.10–1.12) | 1.23 (1.22–1.25) |
| **Diagnosis CD4** |  |  |  |  |
| 0-200 cells/mm^3^ | 96 890 | 21 829 (23%) |  |  |
| 201-350 cells/mm^3^ | 120 538 | 29 323 (24%) | 1.25 (1.23–1.27) | 1.12 (1.10–1.13) |
| 351-500 cells/mm^3^ | 60 501 | 16 894 (28%) | 1.37 (1.35–1.39) | 1.16 (1.14–1.18) |
| 500+ cells/mm^3^ | 66 676 | 20 656 (31%) | 0.23 (0.23–0.23) | 1.28 (1.26–1.30) |
| **Diagnosis setting** |  |  |  |  |
| Hospital | 25 163 | 8 137 (32%) | 1.36 (1.34–1.38) | 1.35 (1.32–1.37) |
| PHC VTP | 39 088 | 12 007 (31%) | 1.24 (1.22–1.26) | 1.23 (1.21–1.25) |
| PHC TB | 57 488 | 14 741 (26%) | 1.02 (1.00–1.03) | 1.14 (1.12–1.16) |
| PHC other | 234 053 | 56 734 (24%) |  |  |
| **Years since diagnosis** |  |  |  |  |
| <5 years | 102 698 | 32 289 (31%) | 1.36 (1.34–1.37) | 1.27 (1.25–1.29) |
| 5 to 10 years | 121 080 | 31 952 (26%) | 1.00 (0.00–0.00) | 1.16 (1.14–1.18) |
| >10 years | 120 827 | 24 461 (20%) |  |  |
| **Age at analysis date** |  |  |  |  |
| 15-24 years | 18 401 | 6 423 (35%) | 1.80 (1.76–1.84) | 1.66 (1.63–1.69) |
| 25-34 years | 85 589 | 29 031 (34%) | 1.77 (1.75–1.80) | 1.29 (1.27–1.31) |
| 35-44 years | 129 742 | 32 157 (25%) | 1.32 (1.30–1.33) | 1.00 (0.00–0.00) |
| 45-54 years | 77 798 | 14 460 (19%) |  |  |
| ≥ 55 years | 33 075 | 6 631 (20%) | 1.13 (1.11–1.16) | 0.13 (0.13–0.14) |
| **Total** | **344 605** |  |  |  |
