# Supplementary material for: Contemporary Disengagement From Antiretroviral Therapy in the Western Cape, South Africa: A Cross‐Sectional Study
Source: J Int AIDS Soc. 2026 May 18;29(5):e70124. doi: 10.1002/jia2.70124 (PMC13181324; doi:10.1002/jia2.70124)
Supplement: Supplementary file 4 — Supporting Table S4: Estimated proportion of the entire study population (N = 494,071) who would need to be targeted (classified as high risk) to identify a target proportion of those disengaged (not on ART), using the full model with multiple imputation for missing CD4 categories. [file JIA2-29-e70124-s004.docx]

| **Target proportion of disengaged (not on ART)** | **Proportion needed to be targeted (classified as high risk)** |
| --- | --- |
| **50%** | 62% |
| **60%** | 72% |
| **70%** | 80% |
| **80%** | 88% |
| **90%** | 94% |
| **95%** | 97% |
