# Supplementary figures and images for: Contemporary Disengagement From Antiretroviral Therapy in the Western Cape, South Africa: A Cross‐Sectional Study
Source: J Int AIDS Soc. 2026 May 18;29(5):e70124. doi: 10.1002/jia2.70124 (PMC13181324; doi:10.1002/jia2.70124)

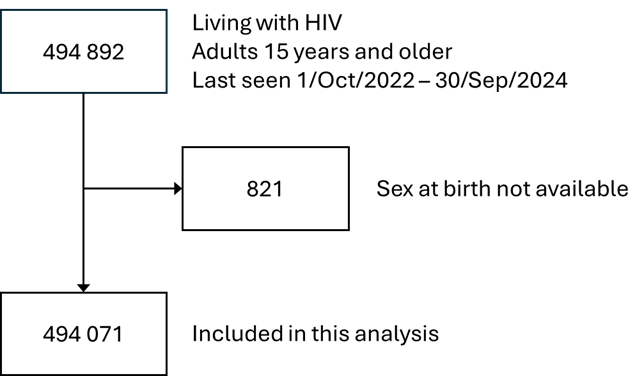

Supplement: Supplementary file 5 — Supporting Figure S1: Enumerated individuals known to the health services to be living with HIV in the Western Cape included in the analysis, excluding those without data on sex at birth. [file JIA2-29-e70124-s001.png]
